# Cryo-EM reveals the structural basis of long-range electron transport in a cytochrome-based bacterial nanowire

**DOI:** 10.1038/s42003-019-0448-9

**Published:** 2019-06-19

**Authors:** David J. Filman, Stephen F. Marino, Joy E. Ward, Lu Yang, Zoltán Mester, Esther Bullitt, Derek R. Lovley, Mike Strauss

**Affiliations:** 1 Department of Biological Chemistry and Molecular Pharmacology, Harvard Medical School, 240 Longwood Avenue, Boston, MA 02115 USA; 2 German Federal Institute of Risk Assessment, Unit Bacterial Toxins, Department of Biological Safety, Max-Dohrn-Str. 8–10, Berlin, 10589 Germany; 3 Department of Microbiology and Institute for Applied Life Sciences, University of Massachusetts, Amherst, MA 01002 USA; 4 National Research Council Canada, Montreal Road Ottawa, ON 1200 Canada; 5 Department of Physiology & Biophysics, Boston University School of Medicine, 700 Albany Street, Boston, MA 02118 USA; 6 Department of Anatomy and Cell Biology, McGill University, 3640 University St, Montreal, H3A 2B2 QC Canada

**Keywords:** Cellular microbiology, Bioenergetics, Soil microbiology

## Abstract

Electrically conductive pili from *Geobacter* species, termed bacterial nanowires, are intensely studied for their biological significance and potential in the development of new materials. Using cryo-electron microscopy, we have characterized nanowires from conductive *G. sulfurreducens* pili preparations that are composed solely of head-to-tail stacked monomers of the six-heme C-type cytochrome OmcS. The unique fold of OmcS — closely wrapped around a continuous stack of hemes that can serve as an uninterrupted path for electron transport — generates a scaffold that supports the unbranched chain of hemes along the central axis of the filament. We present here, at 3.4 Å resolution, the structure of this cytochrome-based filament and discuss its possible role in long-range biological electron transport.

## Abstract (web summary)

David Filman et al. present the cryo-EM structure of an electrically conductive filament from *Geobacter sulfurreducens*, termed bacterial nanowires. They find that these structures are composed of a bacterial cytochrome, and suggest a possible role in long-range biological electron transport.

## Introduction

Microbially produced protein nanowires are of profound interest due to their proposed role in global carbon and metal cycling and promise for myriad practical applications^1–4^. Among the most thoroughly characterized are the electrically conductive pili (e-pili) of *Geobacter* species, which comprises pilin protein monomers^5–7^. *Geobacter* e-pili enable long-range (µms) extracellular electron transfer to Fe(III) minerals and other microbial species, which is important in the biogeochemistry of diverse anaerobic soils and sediments^3,8^. *Geobacter* species are among the most effective microbes in converting organic compounds into electricity, because they can form thick electrically conductive e-pili biofilms^9,10^. e-Pili purified from cells show potential as sustainably produced protein nanowires for electronics, as their properties can readily be tuned with genetic modifications to the pilin monomer, they can be incorporated into polymers to produce composite electronic materials, and they have dynamic sensing properties^3,4,11^. Other bacteria produce e-pili from pilin monomers^12,13^ and the archaeon *Methanospirillum hungatei* expresses an electrically conductive protein filament from archaellin monomers^14^.

Both e-pili and multi-heme outer-surface cytochromes have been implicated as being important for *Geobacter’s* long-range extracellular electron exchange with other cells and soil minerals^8,15^. Determining their respective contributions has been complicated by the previously reported association of OmcS, a cytochrome known to participate in some forms of extracellular electron exchange, with conductive filaments^16^. Immunogold labeling with anti-OmcS antibodies showed staining of thick filaments in pilus preps from *G. sulfurreducens*, leading Leang et al. to propose that OmcS may fulfill a functional role in directly modulating conductivity^17^. OmcS is an abundant component in preparations in which filaments are separated from cells^18^. In pursuing investigation of these possibilities, we focused on the thick filaments in our e-pili preparations and have now solved the structure of these filaments by cryo-electron microscopy (cryo-EM). The 3D particle reconstructions of segments picked exclusively from thick filaments resulted in density that cannot be fit by any combination of PilA pilin monomers, but is perfectly fit by OmcS alone. We conclude that these thick filaments are not simply associated with OmcS, but rather consist of OmcS monomers, thereby representing the first example, to our knowledge, of nanowires composed entirely of a bacterial cytochrome.

## Results

### Nanowire filament identified as OmcS

**Fig. 1:**
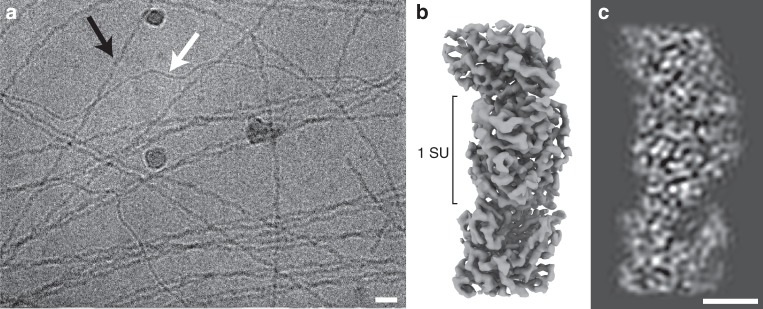
**a** Cryo-electron micrograph of filaments isolated from Geobacter *sulfurreducens*, thicker OmcS filaments (black arrow), and thinner (3 nm diameter) filaments (white) (Scale bar: 20 nm). **b** Isosurface representation of cryo-EM reconstruction of the OmcS filament. The approximate dimension of one subunit (SU) along the filament axis is shown. **c** A density representation of a 10 Å thick central slice through the filament. (Scale bar: 2.5 nm)

**Fig. 2:**
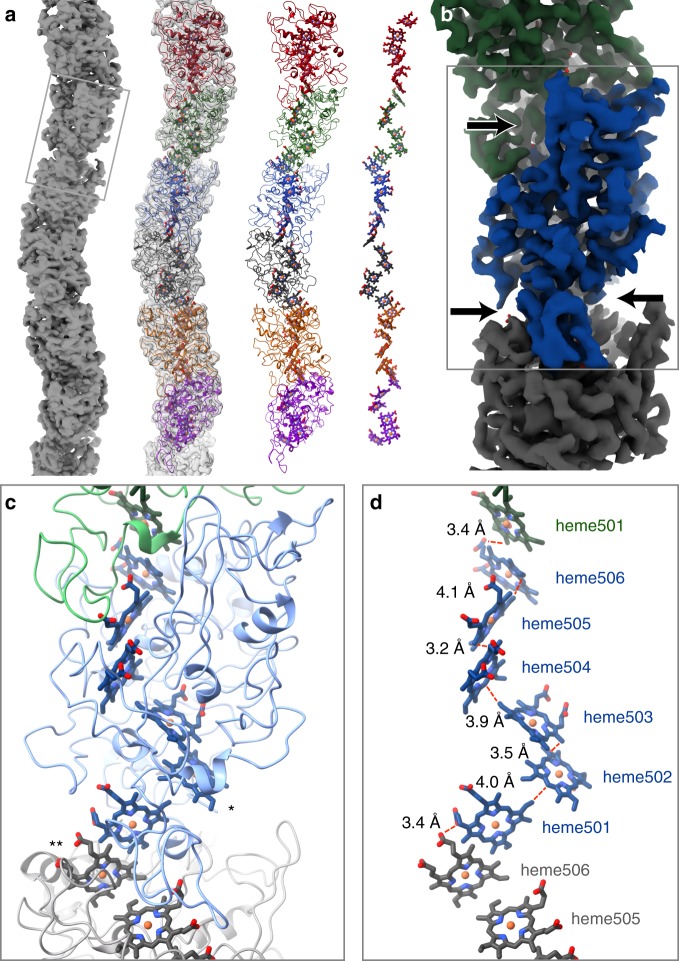
**a** Filamentous OmcS, as seen in projection, snakes back and forth along its length. Left-to-right, it is shown here as a surface rendering, with the model overlayed, as just the model, and depicting only the chain of hemes. **b** A color-coded surface representation showing one OmcS molecule, and with arrows pointing to the narrow interface. The box corresponds to the area shown in the lower panels. **c** A view of the model showing the complex fold of the amino acid chain around the heme groups. Solvent-exposed hemes are labeled as follows: * - heme502, ** - heme506 on the adjacent subunit. **d** Interatomic contact distances between adjacent porphyrins are 4.1 Å or less. The hemes are labeled

**Fig. 3:**
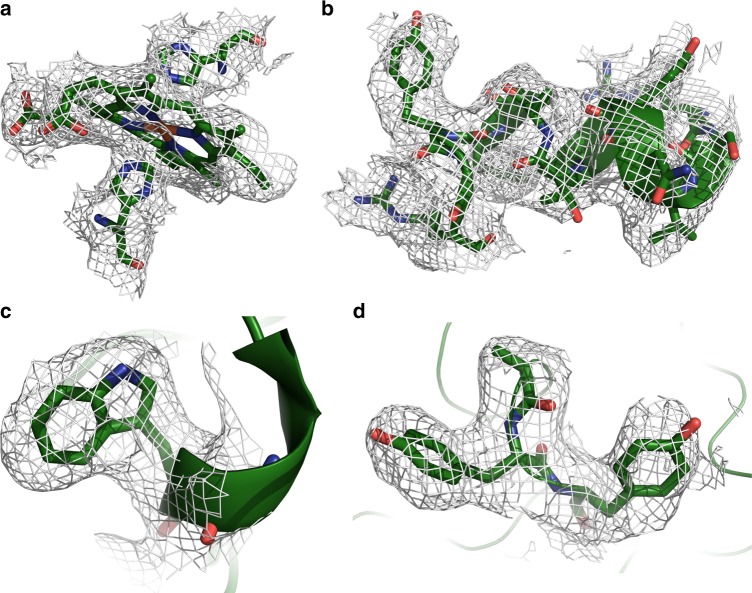
Detailed views of OmcS model within densities. **a** Hemes (in this case Heme503) are coordinated by histidine residues from above and below. **b** OmcS has few secondary structural motifs, but when present, they are well ordered and easily visible, as with this short stretch of alpha helix starting at residue 401. **c** The density for Trp113 is very clear. **d** Bends in the main chain are well resolved, as are large side chains such as Tyr409 and Tyr410

Preparations of conductive e-pili from *G. sulfurreducens* routinely containing multiple filaments with distinct morphological characteristics. In addition to the ca. 3-nm-diameter filaments, presumably composed of the PilA pilin, thicker (ca. 4 nm) filaments are a prominent prep component (Fig. 1a). We pursued structural studies of these 4-nm, ostensibly OmcS-associated, filaments in an attempt to define their interaction with OmcS and determine its contribution to their conductive properties. Individual filament segments were extracted from cryo-EM images of conductive e-pili preps and, after analysis, the resulting 3D reconstruction (Fig. 1b, c) produced a map at 3.4 Å resolution (Table 1; Supplementary Fig. 1) with extensive contiguous polypeptide density that could not satisfactorily fit with any combination of PilA monomers and also showed multiple regions of density along the filament providing excellent fits to c-type heme molecules (Figs. 2a and 3a). We concluded that PilA could neither harbor these hemes due to its lack of characteristic cysteines that covalently bind to c-type hemes nor any histidine residues that commonly coordinate the iron center (Fig. 3a); additionally, there were clear densities for aromatic side chains (Fig. 3b–d), such as tryptophan (Fig. 3c), that are also absent from the PilA sequence. In subsequent fitting attempts using the sequences of several related *Geobacter* cytochromes, only that of OmcS provided a near ideal fit to the density, incorporating the full OmcS sequence corresponding to the 47.5 Å filament repeat and accounting for all 6 hemes per OmcS monomer (Fig. 2c). As there is no density in the maps not accounted for by OmcS, we conclude that this filament is composed only of OmcS.

**Table 1:** Cryo-EM data collection, refinement and validation statistics

	OmcS filament (EMD-9357) (PDB 6NEF)
Data collection and processing	
Magnification	130000 (EFTEM)
Voltage (kV)	300
Electron exposure (e–/Å^2^)	50
Defocus range (μm)	1–3.5
Pixel size (Å)	1.06
Symmetry imposed	Helical: rise: 48 Å rotation: 83°
Particle images (no.)	462922
Map resolution (Å)	3.4
FSC threshold	0.143
Refinement	
Model resolution range (Å)	180–3.33
Map sharpening *B* factor (Å^2^)	−117.08
Model composition	
Non-hydrogen atoms	9819
Protein residues	1221
Ligands	18 hemes, 3 Mg^2+^
Phase error (°)	28.72
*B* factors (Å^2^)	2–445
Protein	107
Ligand	65
R.m.s. deviations	
Bond lengths (Å)	0.014
Bond angles (°)	1.85
Validation	
Clashscore	33.58
Poor rotamers (%)	0
Ramachandran plot	
Favored (%)	72.1
Allowed (%)	24.2
Disallowed (%)	3.7

### OmcS filament architecture

The OmcS monomer from *G. sulfurreducens* is a six-heme c-type cytochrome (UniProt Q74A86) and the OmcS fiber is a helically symmetric arrangement containing hundreds of monomers. Each monomer repeat extends ~4.8 nm along the fiber axis, is rotated 83° and is ~4 nm wide except at the intermonomer interfaces, where the fiber narrows substantially (Figs. 1b and 2a, b). At the core of the fiber is a continuous, unbranched stack of hemes (Fig. 2a, d).

Each heme has two nearest neighbors, one above and one below. Successive hemes, approaching edge-to-edge, alternate between lying parallel and perpendicular to the previous heme (Fig. 2d). The interatomic contact distances are all ≤4.1 Å (see Supplementary Table 1). This proximity suggests a mechanism for long-range electrical conduction, but leaves open the question of how charges enter and exit the nanowire.

The OmcS sequence includes six Cys-x-x-Cys-His motifs, with both Cys sulfur atoms covalently attached to heme substituents, and His as one of the axial ligands (Fig. 3a). The stacking order of hemes along the fiber axis coincides with the order of the Cys-x-x-Cys-His motifs in the sequence. This exact correspondence has been seen in some (e.g., PDB 3ov0^19^), but not in all (e.g., PDB 1z1n), multiheme cytochromes (Supplementary Fig. 2). All 12 heme-axial ligands are histidine side chains, with 11 coming from the same OmcS subunit, and 1 (attached to heme505) from a neighboring molecule.

The main chain density for the OmcS protein (residues 26–432) is continuous and complete, with distinct bends at most of the alpha carbon positions (Fig. 3d), though the orientation of the peptide plane is not always obvious. Side chain densities, when well ordered, are consistent with the OmcS sequence (Fig. 3b–d). However, some side chains, scattered throughout the sequence, are poorly ordered, as reflected by high-temperature factors in the atomic model (Supplementary Fig. 3). The protein wraps around the central heme stack, nearly completely shielding the hemes from solvent. Notable exceptions, which are solvent-exposed edge-on, include heme502 (from one OmcS subunit) and heme506 (from the adjacent subunit), which are both located near the narrow, intersubunit interfaces (on opposite sides of the fiber) (Fig. 2b, c). These two sites could potentially serve to conduit electrons into or out of the filament.

The heme stack is integral to both the monomer and fiber structures, and dictates the folding pattern of the surrounding 407-residue protein chain, with only ~100 residues involved in forming regular secondary structure, according to KSDSSP^20^; the overall fold is unique among solved protein structures (by VAST search, https://www.ncbi.nlm.nih.gov/Structure/VAST/vastsearch.html).

Each OmcS monomer also includes one octahedrally coordinated metal, tentatively identified as Mg^++^ based on its density level and experiment (see Supplementary Table 2), whose biological significance is unclear. Two of its ligand positions are occupied by acidic substituents of heme504 (see Supplementary Fig. 4c).

## Discussion

The unexpected filamentous assembly of a c-type cytochrome in our structure suggests new possibilities for long-range biological electron transfer. Heme-to-heme electron transport occurs within individual multiheme c*-*type cytochromes and electron transfer between two multiheme cytochromes held within a barrel protein is possible^21^, although nanowires comprised solely of multiple cytochromes have not been observed. The cytochrome-rich membrane extensions of *Shewanella oneidensis*, when dried, are electrically conductive^22,23^. However, under physiologically relevant hydrated conditions, gaps between the membrane associated cytochromes prevent long-range cytochrome-to-cytochrome electron hopping/tunneling^1,24^.

Several lines of evidence attest to the importance of OmcS on the extracellular surface of *Geobacter* cells. It is present on the outer membranes of both wild-type cells^17^ and cells unable to produce e-pili^25^. OmcS deletion strains are deficient in Fe(III) oxide reduction, but expression *in trans* rescues the phenotype^26^, and strains overexpressing OmcS show enhanced electron transfer between *Geobacter* species^27^. This suggests that OmcS filaments may increase the coverage of the cell surface with redox-active moieties beyond that possible with other surface cytochromes that are connected to the periplasm through outer-membrane conduits^15^. OmcS filaments may also provide an electron path between surface cytochromes electrically connected to the periplasm and e-pili for long-range extracellular electron transfer. Additional studies are warranted to determine if nanowires comprised hemes are present in other microbes and to explore the possibilities of employing heme-based nanowires as a sustainably produced material for electronic devices.

## Methods

### Nanowire isolation

As previously described^18^, biofilms that were grown on potentiostat-poised electrodes as the electron acceptor were gently scraped from the graphite surface with a plastic spatula, and suspended in a buffer of: 20.02 mM morpholinepropanesulfonic acid, 4.35 mM NaH_2_PO_4_ · H_2_O, 1.34 mM KCl, 85.56 mM NaCl, 1.22 mM MgSO_4_ · 7H_2_O, and 0.07 mM CaCl_2_ · 2H_2_O. The cells were pelleted with a centrifuge and then resuspended in ethanolamine buffer (150 mM, pH 10.5). The suspended cells were placed in a Waring blender at low speed for 1 min to shear filaments from the cells. The cells were separated from the filaments by centrifugation at 13000 × g, leaving filaments in the supernatant. Ammonium sulfate was added to the supernatant to 10% final concentration to precipitate filaments. After an overnight incubation, the sample was centrifuged at 13000 × g. The supernatant was removed and the precipitate was resuspended in ethanolamine buffer. The material was then centrifuged at 23000 × g and the resulting supernatant was treated with ammonium sulfate (10% final concentration). The precipitate derived after centrifugation at 13000 × g was resuspended in ethanolamine buffer and then dialyzed against deionized water to remove buffer constitutents.

### Preparation of cryo-EM samples, data collection and processing

Samples for cryo-EM were added to glow discharged R2/2 Quantifoil grids and plunge-frozen on a Vitrobot Mk IV (ThermoFisher). Imaging was carried out on a Titan Krios (ThermoFisher) equipped with a Bioquantum K2 GIF (Gatan Inc) using a magnification corresponding to a 1.06 Å calibrated pixel size. The 45-frame acquisitions were acquired with SerialEM^28^ using counting mode and accumulating a total dose of 50 e^-^Å^−2^ over 15 s. Frame alignment was done in MotionCor2, and all subsequent processing steps were carried out in Relion-3^29^. Filaments were manually selected and overlapping segments were extracted, and refined to produce a 3.9 Å map. 2D and 3D classification was carried out, but proved unnecessary, due to the homogeneity of the filaments selected. After particle polishing, the final reconstruction was consistent with a 3.4 Å map (as determined by FSC 0.143; Table 1; Supplementary Fig. 1).

### Model building and refinement

Attempts at model building with the initial map (resolution ca. 3.9 Å) were performed exclusively with the 61-amino acid PilA sequence and yielded no convincing fits to the density. Further *de novo* attempts starting from poly-alanine chains yielded neither repeated sequence modules nor any stretch with substantial homology to that of PilA. Early inspection of the reconstruction clearly showed a line of metal sites running along the fiber axis and a continuous stack of six porphyrins per helical subunit (Supplementary Fig. 4). Detailed atomic models for the protein were added to the reconstruction, repeatedly rebuilt using COOT^30^ and SPDBV^31^, and refined using Refmac5^32^ and Phenix.autobuild^33^. Subsequent efforts to convincingly identify one or more copies of PilA in the map were unsuccessful. As the map resolution improved, several density patterns were identified that were clearly incompatible with the PilA sequence, which lacks Trp, His, and Cys, has no adjacent aromatic residues, and is predicted to have substantial helical content. To discover what else might be present, atomic models were prepared with arbitrary sequences closely resembling the density. Once sequence database searches with the arbitrary sequences, using psi-Blast^34^, found similarities to a number of related six-heme c-type cytochromes, including OmcT and OmcJ (uniprot: Q74A87 and Q74FA8, respectively), we focused, among these, upon OmcS, which was known to be abundant in the fiber sample^17^. An atomic model with the OmcS sequence was then fitted to the map, and produced a convincing visual fit, accounting for all of the calculated density.

In phenix.refine, a stereochemically restrained atomic model (of three adjacent OmcS subunits), having RMS bond length deviations of 0.014 Å, yielded an R-factor of 24.0%, and an estimated phase error of 28.7°, when compared with the Fourier transform (amplitudes and phases) of the corresponding portion of the map (see Table 1).

### Reporting summary

Further information on research design is available in the Nature Research Reporting Summary linked to this article.

## Supplementary information

Supplementary Information

Reporting Summary

## Data Availability

The map and model are available under accession numbers: EMD: EMD-9357, PDB: 6NEF, respectively.
