# Supplementary material for: Cryo-EM reveals the structural basis of long-range electron transport in a cytochrome-based bacterial nanowire
Source: Commun Biol. 2019 Jun 19;2:219. doi: 10.1038/s42003-019-0448-9 (PMC6584659; doi:10.1038/s42003-019-0448-9)
Supplement: Supplementary file 1 — Supplementary Information [file 42003_2019_448_MOESM1_ESM.pdf]

Supplementary Figure 1

Fourier Shell Correlation for cryoEM reconstruction of OmcS filament.

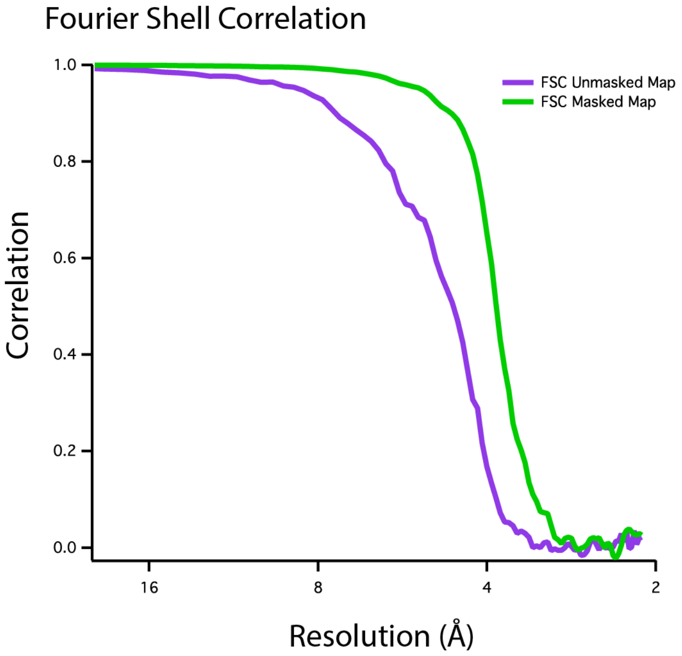

Supplementary Figure 2

OmcS in relation to other multi-heme cytochromes. 1z1n is a branched 16-heme cytochrome from Desulfovibrio gigas[1], while the others are from Geobacter sulfurreducens: 3ov0 – 12-heme cytochrome c [2], 3bxu – 3-heme cytochrome c [3], 1os6 – 3-heme cytochrome c7 [4], 3h33 – 3-heme cytochrome c7 [5], OmcS (pdb: 6nef) – 6-heme cytochrome.

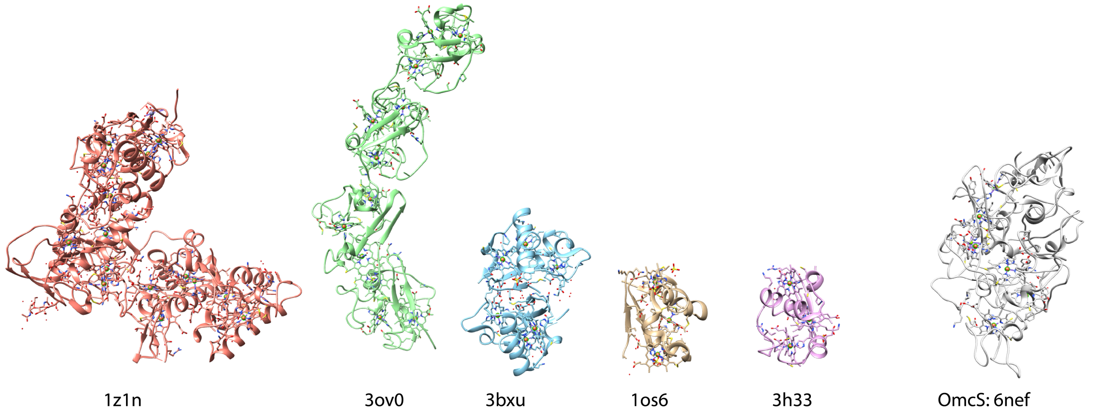

Supplementary Figure 3

OmcS shown with the thickness of the tube related to the b-factor of the model in that area. This reflects the areas in the model where the fit to the density contained the most uncertainty.

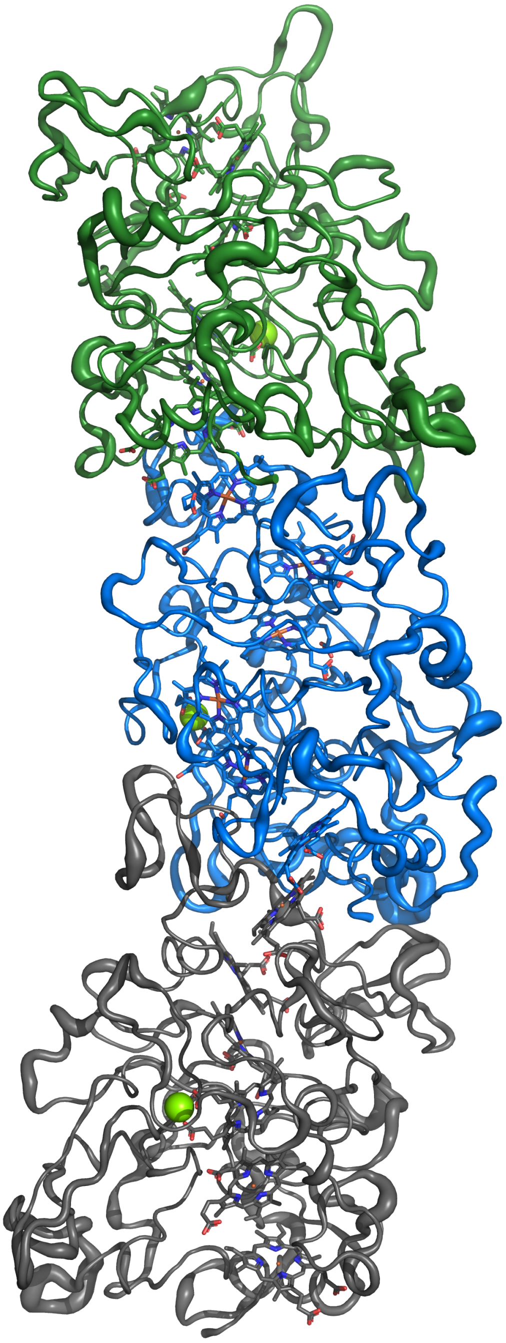

Supplementary Figure 4

(A) The heme centers overlayed on the density map at high threshold (coloured purple for clarity). (B) at lower threshold the heme centers cover the core of each porphyrin. The histidines that coordinate the iron are evident as densities perpendicular to the porphyrin planes. (C) The putative magnesium ion is coordinated by heme504.

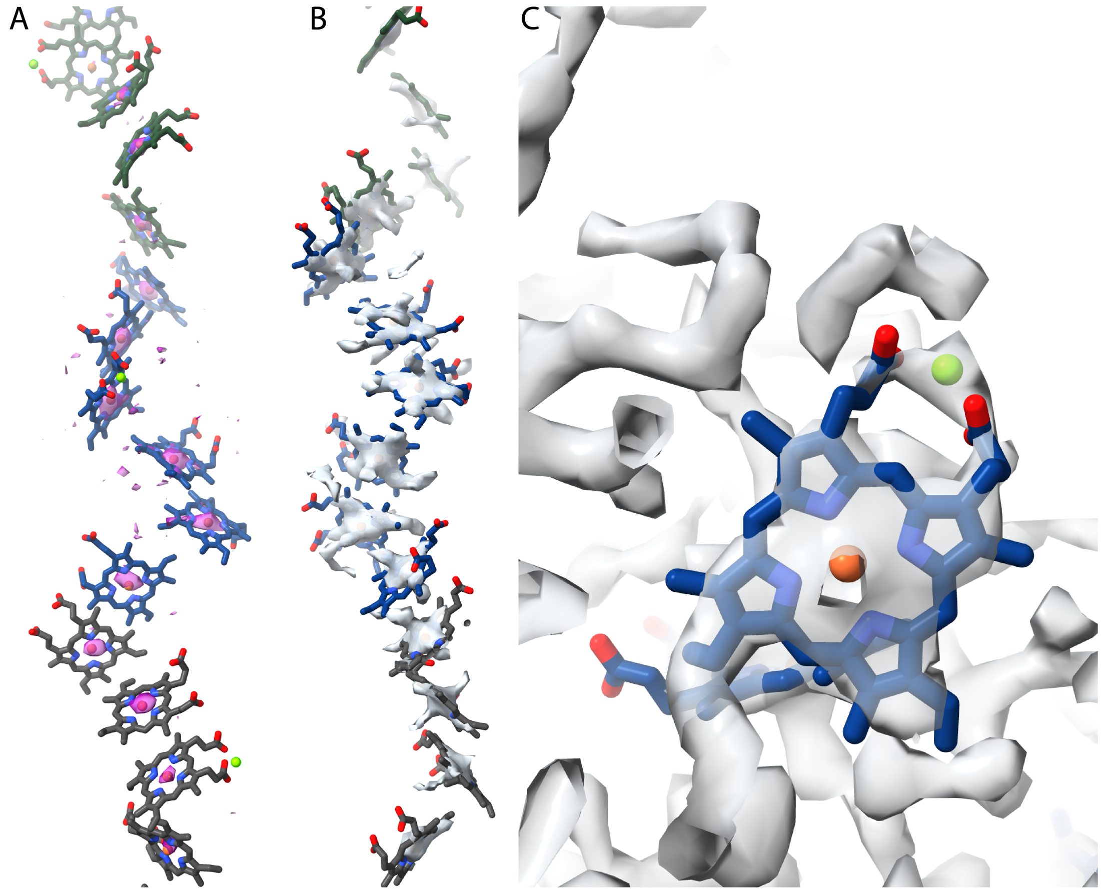

Supplementary Table 1

In Supplementary Table: Closest interatomic contacts between neighbouring hemes, inter-iron distances, and angles between heme planes.

Heme	Atom	Heme	Atom	Distance (Å)	Fe-Fe Distance (Å)	Inter-heme angle [degrees]
501	CBC	502	CHC	4.0	13.0	71.4
502	C3A	503	CMA	3.5	9.3	176.9
503	CBC	504	CHC	3.9	11.2	67.6
504	C2A	505	CMA	3.2	9.3	175.1
505	CBC	506	CAB	4.1	11.3	96.5
506	O2A	501	CAA	3.4	9.0	168.8

Supplementary Table 2: Inductively coupled plasma mass spectrometry (ICP-MS)

	Concentration, mg/kg													
**Sample Name**	**Fe**	**SD (Fe)**	**Mg**	**SD (Mg)**	**K**	**SD (K)**	**Ca**	**SD (Ca)**	**Mn**	**SD (Mn)**	**Zn**	**SD (Zn)**	**Sr**	**SD(Sr)**
Sample W	<0.005		<0.01		<0.02		<0.01		<0.002		<0.002			<0.002
Sample B	0.049	0.005	<0.01		0.12	0.01	<0.01		0.0029	0.0003	<0.002			<0.002
Sample B+W	0.104	0.003	<0.01		<0.02		<0.01		0.0028	0.0003	<0.002			<0.002
Sample G Sulf Pili	1.47	0.02	0.61	0.02	0.41	0.02	0.24	0.01	0.0125	0.0006	0.01	0.001	0.0046	0.0005

sample w = milli-Q water directly from our system

sample b = 150mM Ethanolamine, pH 10.5 made using Ethanolamine-HCl plus NaOH.

sample w + b = 150mM Ethanolamine, pH 10.5 dialyzed against milli-Q water

References

1 Santos-Silva, T. *et al.* Crystal structure of the 16 heme cytochrome from Desulfovibrio gigas: a glycosylated protein in a sulphate-reducing bacterium. *J. Mol. Biol.* **370**, 659-673, doi:10.1016/j.jmb.2007.04.055 (2007).

2 Pokkuluri, P. R. *et al.* Structure of a novel dodecaheme cytochrome c from Geobacter sulfurreducens reveals an extended 12 nm protein with interacting hemes. *J. Struct. Biol.* **174**, 223-233, doi:10.1016/j.jsb.2010.11.022 (2011).

3 Morgado, L. *et al.* Structural insights into the modulation of the redox properties of two Geobacter sulfurreducens homologous triheme cytochromes. *Biochim. Biophys. Acta* **1777**, 1157-1165, doi:10.1016/j.bbabio.2008.04.043 (2008).

4 Pokkuluri, P. R., Londer, Y. Y., Duke, N. E., Long, W. C. & Schiffer, M. Family of cytochrome c7-type proteins from Geobacter sulfurreducens: structure of one cytochrome c7 at 1.45 A resolution. *Biochemistry* **43**, 849-859, doi:10.1021/bi0301439 (2004).

5 Pokkuluri, P. R. *et al.* Structural characterization of a family of cytochromes c(7) involved in Fe(III) respiration by Geobacter sulfurreducens. *Biochim. Biophys. Acta* **1797**, 222-232, doi:10.1016/j.bbabio.2009.10.007 (2010).
